# Specific and rapid reverse assaying protocol for detection and antimicrobial susceptibility testing of *Pseudomonas aeruginosa* based on dual molecular recognition

**DOI:** 10.1038/s41598-021-90619-3

**Published:** 2021-05-27

**Authors:** Yong He, Hang Zhao, Yuanwen Liu, He Zhou

**Affiliations:** 1 Department of Pharmacy, Affiliated Hospital of Zunyi Medical University, Zunyi, 563000 China; 2 Zunyi Institute for Food and Drug Control, Zunyi, 563000 China

**Keywords:** Bioanalytical chemistry, Medical and clinical diagnostics

## Abstract

The worldwide emergence and spread of antimicrobial resistance is accelerated by irrational administration and use of empiric antibiotics. A key point to the crisis is a lack of rapid diagnostic protocols for antimicrobial susceptibility testing (AST), which is crucial for a timely and rational antibiotic prescription. Here, a recombinant bacteriophage tail fiber protein (TFP) was functionalized on magnetic particles to specifically capture *Pseudomonas aeruginosa*, while fluorescein isothiocyanate-labeled-magainin II was utilized as the indicator. For solving the magnetic particles’ blocking effects, a reverse assaying protocol based on TFP recognition was developed to investigate the feasibility of detection and AST of *P. aeruginosa*. *P. aeruginosa* can be rapidly, sensitively and specifically detected within 1.5 h with a linear range of 1.0 × 10^2^ to 1.0 × 10^6^ colony forming units (CFU)⋅mL^−1^ and a detection limit of 3.3 × 10 CFU⋅mL^−1^. Subsequently, AST results, which were consistent with broth dilution results, can be obtained within 3.5 h. Due to the high specificity of the TFP, AST can actually be conducted without the need for bacterial isolation and identification. Based on the proof-of-principle work, the detection and AST of other pathogens can be extended by expressing the TFPs of their bacteriophages.

## Introduction

Antimicrobial resistance (AMR) is a worldwide health crisis resulting in growing economic burden and increasing mortality^1,2^. The most important strategies to minimize AMR are the prudent antibiotic use and the development of new antibiotics^3,4^. For the correct antibiotic use, the development of rapid and accurate diagnostic protocols for antimicrobial susceptibility testing (AST) facilitates the timing of prescription of effective antibiotics. Therefore, numerous efforts are exerted on developing rapid, sensitive and acute detection and AST for bacterial infections.

Traditional bacterial growth-based protocols are considered as the gold standard methods for bacterial detection and AST. These protocols ideally need to show repeatability, high standardization and good reliability. However, the isolation and identification procedures for bacterial detection usually require a tedious process of 24–48 h^5^. The subsequent broth dilution or disk diffusion tests for AST require bacterial culturing with given concentrations of various antibiotics, which demands almost another 24–48 h^6,7^. Lack of timely results of bacterial AST results in frequent empiric antibiotic therapy, causing irrational antibiotic use and the development of AMR^8^. Therefore, some other bacterial growth-based protocols are reported to aim at shortening the time of AST, such as microscopy detection^9–11^, electrochemical sensor^12–14^, phase-shift spectroscopy detection^15^, fluorescent detection^16^, microfluidic devices based on the slipchip technique^17^, and surface-enhanced raman scattering^18^. Nevertheless, due to their lack of capacity of identifying the given bacterial species, they also require time-consuming pretreatment procedures for bacterial culturing, isolation and identification. Polymerase chain reaction-based protocols gradually attract attention for bacterial detection and AST due to the advantages of rapidity, sensitivity and because it is a culture-free process^20–22^. However, they suffer from the prerequisite of precise resistant gene information and frequent gene mutation.

With the increasing global AMR, bacteriophages have regained interest as antimicrobial agents since they are the natural enemies of the bacteria. Bacteriophages highly specifically (sometimes up to the strain level) recognize their target bacteria even in harsh environments. A typical lifecycle of a virulent bacteriophage involves the following steps: specific adsorption on the bacterial cell wall, deoxyribonucleic acid (DNA) injection into the bacterial cell, the formation of new phage particles and the lysis of bacteria cell for releasing its progeny. Bacteriophage receptor binding proteins such as tail fiber proteins (TFP), tailspike proteins (TSP) and the endolysin are the essential recognition elements which are responsible for adsorption, injection and lysis, respectively^23^. Therefore, receptor binding proteins are the ideal molecular recognition attributes for a high specificity, robustness, good anti-interference capability and universality to each bacterium^23^. Unfortunately, just like the bacteriophage entity as a whole, the endolysin and TSP both have an inherent lytic activity which could be unfavorable for bacterial capture and sample manipulation.

Previously, we showed that the TFP (NCBI accession number YP 007236480.1) of *Pseudomonas aeruginosa* phage PaP1, recombinantly produced in *Escherichia coli*, can specifically recognize *P. aeruginosa*. This recombinant TFP can specifically recognize *P. aeruginosa* without displaying a lytic activity. To investigate the application of this TFP in AST, TFP-functionalized magnetic particles (MPs) were utilized to specifically capture *P. aeruginosa*, and fluorescein isothiocyanate (FITC) labeled magainin II was utilized as the fluorescent tracer. A reverse assaying protocol (RAP) combined with magnetic separation was developed to conduct specific, rapid and sensitive detection and AST of *P. aeruginosa*. In the RAP, quantitative excess FITC labeled magainin II is mixed with a suspension of *P. aeruginosa*. After *P. aeruginosa* is captured and magnetically separated by TFP-functionalized MPs, the supernatant solution of remaining fluorescent tracer is utilized to quantify *P. aeruginosa*. The change of *P. aeruginosa* concentrations under various antibiotics can indirectly respond to the susceptibility of *P. aeruginosa* to the antibiotics.

## Experimental setup

### Instrumentations

Scanning electron micrographs (SEM) were recorded by an S-3000 N scanning electron microscope (Hitachi, Japan). Fluorescence (FL) signals were obtained using an Infinite M200 PRO microplate reader (TECAN, Switzerland). FL micrographs were recorded by using a NI − U FL microscope (Nikon, Japan).

### Reagents and materials

Piperacillin/Tazobactam (PIP/TAZ, catalog number: P1780/YZ-130511), ceftazidime (CAZ, catalog number: C9730), tobramycin (TOB, catalog number: T8810), gentamicin (GEN, catalog number: IG0770) and levofloxacin (LVX, catalog number: IL0090) were all purchased from Solarbio Life Sciences (Beijing, China). All the strains of *P. aeruginosa* were obtained from the China Center for Type Culture Collection expect the phage’s host strain PA1. The specificity test bacteria of *E. coli* (GIM 1.223), *Pseudomonas solanacearum* (GIM 1.77), *Salmonella* Typhimurium (GIM 1.237), *Staphylococcus aureus* (GIM 1.644), *Staphylococcus epidermidis* (GIM 1.444), *Streptococcus mutans* (GIM 1.530) were purchased from Guangdong Microbiology Culture Center (Guangdong, China). FITC (catalog number: F106837) labeled magainin II (catalog number: M119006) were obtained from GL Biochem Ltd.. (Shanghai, China). Tetraethyl rhodamine isothiocyanate (TRITC, catalog number: 87918) and gelatin (catalog number: 1288485) came from Sigma-Aldrich (Shanghai, China). AffiAmino MPs (catalog number: 1003) were purchased from Lab on a Bead AB (Uppsala, Sweden) to which activation buffer and blocking buffer were provided. Both human urine collected from the authors and rat plasma given by the Key Laboratory of Basic Pharmacology of Zunyi Medical University were used as the common matrices. Luria − Bertani (LB) broth consisted of 10 g⋅L^−1^ NaCl, 5 g⋅L^−1^ yeast extract and 10 g L^−1^ tryptone. Washing buffer was composed of 10 mM phosphate buffer saline (PBS, pH 7.4) and 0.5% tween-20. More information on the production of recombinant TFP can be found in the supplementary material.

### Bacterial culturing and counting

Strains of *P. aeruginosa* and other bacteria were cultured in 30 mL of LB broth with continuous shaking at 80 rotations per minute (rpm) and 37 °C under aerobic conditions until the optical density at 600 nm (OD_600_) reached 1.0. For bacterial detection, 10 mM PBS (pH 7.4) was utilized to serially dilute the bacteria to reach the target concentration. The bacterial concentrations were evaluated by plating.

### Preparation procedure of TRITC-labeled TFP

One milliliter of TFP solution in the PBS buffer (10 mM, pH 7.4) at the concentration of 1.0 mg⋅mL^−1^ was slowly mixed with 1.0 mL of TRITC solution at 3.0 mg⋅mL^−1^ dissolved in dimethyl sulfoxide, followed by a 12-h reaction at 4 °C. Subsequently, NH_4_Cl solution was used to stop the reaction. Finally, the solution was dialyzed with 10 mM PBS (pH 7.4) for 48 h at room temperature.

### Fluorescent microscope image of stained P. aeruginosa

One milliliter of *P. aeruginosa* suspension at 1.0 × 10^6^ CFU⋅mL^−1^ was added to 200 μL of FITC labeled magainin II at 20.0 μg⋅mL^−1^ and an equal volume of TRITC labeled TFP. After a 1-h incubation at room temperature, the suspension was centrifuged at 2500 g and washed thrice, followed by resuspension in 10 mM PBS (pH 7.4). Subsequently, 10 μL of the stained *P. aeruginosa* suspension were observed under a FL microscope with the magnification of 1,000. The excitation wavelengths of TRITC and FITC were 544 nm and 488 nm, respectively, and the emission wavelengths of TRITC and FITC were 570 nm and 525 nm, respectively.

### Preparation procedure of TFP-functionalized MPs

TFP-functionalized MPs were obtained according to the following procedures. Firstly, 100 μL of MPs were dispensed in a test tube, after which the storage solution was removed by magnetic separation. After washing with washing buffer, 100 μL of MPs were resuspended into 1.0 mL of washing buffer. Subsequently, 50 μL of the activation buffer was mixed with the above suspension for 15 min. After the MPs were thoroughly washed, 1.0 mL of TFP solution at 100 μg⋅mL^−1^ was added for a 1-h reaction at room temperature, followed by another thorough washing. Then the residue active sites of MPs were blocked with 80 μL of the blocking buffer and 1% gelatin at room temperature for 45 min. Finally, after washing thrice, the TFP-functionalized MPs were stored in 10 mM PBS (pH 7.4) at 4 °C.

### RAP for P. aeruginosa

One milliliter of *P. aeruginosa* suspension was added to 10 μL of TFP-functionalized MPs for a 45-min incubation at room temperature. After magnetic separation and thorough washing by the washing buffer thrice, the TFP-functionalized MPs were re-suspended into 100 μL of 10 mM PBS (pH 7.4). Subsequently, the suspension was mixed with 100 μL of FITC labeled magainin II at 20.0 μg⋅mL^−1^ for another 45-min incubation at room temperature. Finally, after the MPs complex was separated, the supernatant solution was transferred into a 96-well microplate to obtain the FL signals with an excitation wavelength and emission wavelength of 488 nm and 525 nm, respectively.

### AST of P. aeruginosa

The stock solutions of antibiotics were prepared according to the Performance Standards for Antimicrobial Susceptibility Testing of Clinical and Laboratory Standards Institute (CLSI) M100S (29th Edition). Five hundred microliters of bacterial suspensions were mixed with the same volume of serial concentrations of antibiotic solutions at 37 °C for 2 h. With the above RAP for *P. aeruginosa* detection, the susceptibility of *P. aeruginosa* was assessed by the change of bacterial concentrations which was measured by the FL signals.

## Results and discussion

### The principle of RAP for P. aeruginosa detection

**Figure 1:**
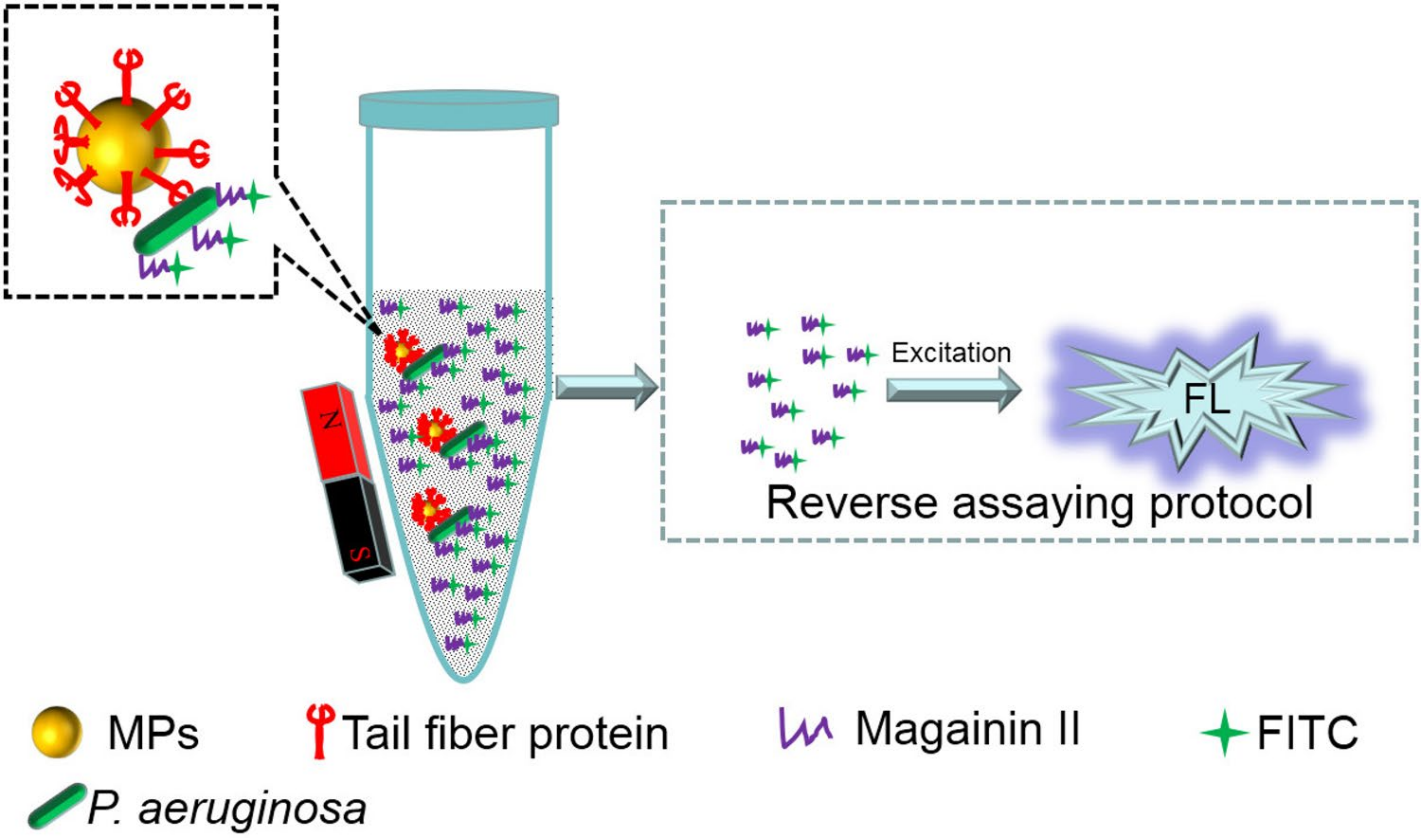
Schematic illustration of RAP for *P. aeruginosa* detection.

As shown in Fig. 1, we developed a RAP for *P. aeruginosa* detection. Recombinant TFP was functionalized on MPs to specifically capture *P. aeruginosa* through a specific interaction between TFP and its receptor on the bacterial cell wall^24,25^. FITC labeled magainin II anchoring on the cytoplasmic membrane of both Gram-positive and Gram-negative bacteria through electrostatic and hydrophobic interactions was utilized as the fluorescent tracer^26,27^. When *P. aeruginosa* was captured by the TFP-functionalized MPs to form a bacteria-MPs complex, quantitative excess FITC labeled magainin II was added to this complex. After FITC-magainin II-bacteria-MPs complex was magnetically separated, the supernatant solution was transferred into the microplate to obtain the FL intensity. The changed values of FL intensity (ΔFL) were utilized to quantify *P. aeruginosa*, in which the ΔFL was defined as: ΔFL = FL_blank sample_ – FL_sample_. Compared to a direct assaying protocol, a RAP can solve the blocking effects of the MPs to the intensity of FITC since the size of MPs is much bigger than that of FITC. When the FITC-magainin II-bacteria-MPs complex was formed, only the FL tracer of the complex on the side of exciting light can be triggered to emit the FL signals.

**Figure 2:**
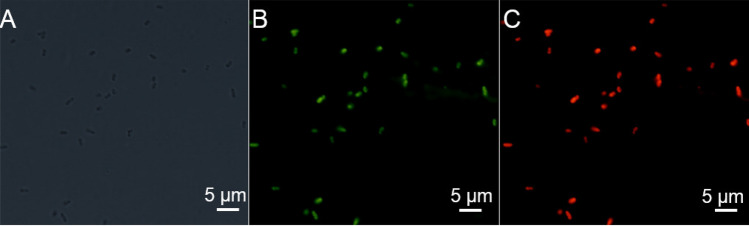
FL microscope image of stained *P. aeruginosa*. (**A**) bright field, (**B**) green FL channel, (**C**) red FL channel.

To assay the coinstantaneous binding capability of TFP and magainin II to *P. aeruginosa*, TRITC labeled TFP and FITC labeled magainin II were simultaneously mixed with *P. aeruginosa* to stain the cells of *P. aeruginosa*. As shown in Fig. 2, red FL from TRITC and green FL from FITC can be both observed on the surface of *P. aeruginosa* cells. This phenomenon demonstrates that TFP and magainin II can simultaneously bind with *P. aeruginosa* at different sites to form the sandwich complex.

### Characterization of capture of by TFP-functionalized MPs

**Figure 3:**
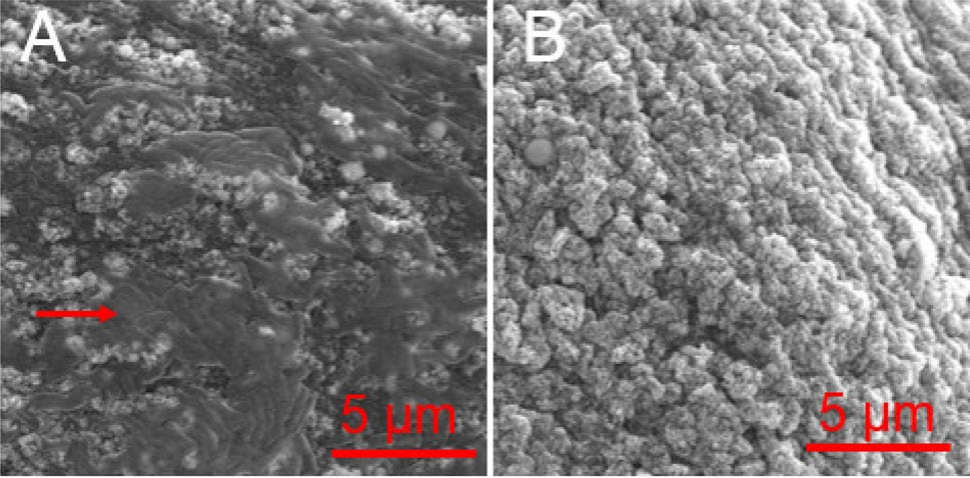
SEM image of (**A**) *P. aeruginosa* captured on the surface of TFP-functionalized MPs and (**B**) bare surface of TFP-functionalized MPs. The red arrow shows the captured *P. aeruginosa*.

To investigate the capture capacity of TFP-functionalized MPs to *P. aeruginosa*, SEM was utilized to observe the capture behavior of TFP-functionalized MPs. As shown in Fig. 3, compared to the bare MPs, *P. aeruginosa* cells were bound and observed on the surface of TFP-functionalized MPs. This demonstrated that after functionalization on MPs, TFP retained its binding capacity to *P. aeruginosa*.

### Condition optimization of P. aeruginosa detection

To enhance the sensitivity of RAP for *P. aeruginosa* detection, the following parameters were evaluated including (1) the amount of TFP-functionalized MPs, (2) the incubation time for *P. aeruginosa* and FITC labeled magainin II and (3) the concentration of FITC labeled magainin II. As illustrated in Figure S1-S3, the values of ΔFL reached an optimum with the following parameters: (1) the use of 10 μL of TFP-functionalized MPs; (2) 45 min incubation time for *P. aeruginosa*; (3) 45 min incubation time for FITC labeled magainin II; and (4) 20.0 μg⋅mL^−1^ of FITC labeled magainin II.

### Detection performance

Under the optimal experimental conditions, this RAP for *P. aeruginosa* detection showed a linear range of 1.0 × 10^2^ to 1.0 × 10^6^ CFU⋅mL^−1^ with a detection limit of 3.3 × 10 CFU⋅mL^−1^. The regression equation was lg *ΔFL* (a.u.) = 1.41 + 0.404 lg *C* (CFU⋅mL^−1^) with a correlation coefficient of 0.9952 (Figure S4). Here, *ΔFL* and *C* represent the changed values of FL intensity and the concentration of *P. aeruginosa*, respectively. The relative standard deviation (RSD) values at low (1.0 × 10^2^ CFU⋅mL^−1^), medium (1.0 × 10^4^ CFU⋅mL^−1^) and high (1.0 × 10^6^ CFU⋅mL^−1^) concentrations were 6.44%, 3.67% and 2.18%, respectively. These results demonstrate RAP showed an acceptable repeatability.

### Specificity

The specificity of RAP was investigated by selecting three Gram-negative bacteria (*E. coli*, *P. solanacearum* and *S.* Typhimurium) and three Gram-positive bacteria (*S. aureus*, *S. epidermidis* and *S. mutans*). The concentrations of these interference bacteria were all 1.0 × 10^5^ CFU⋅mL^−1^ for the specificity investigation. The specificity of the RAP was calculated by the designed interference degree (ID) values of the above interference bacteria in the following equation.

1 $$ ID \, = \Delta FL_{{\text{interference bacteria}}} /\Delta FL_{P. \, aeruginosa} \times 100\% $$

**Figure 4:**
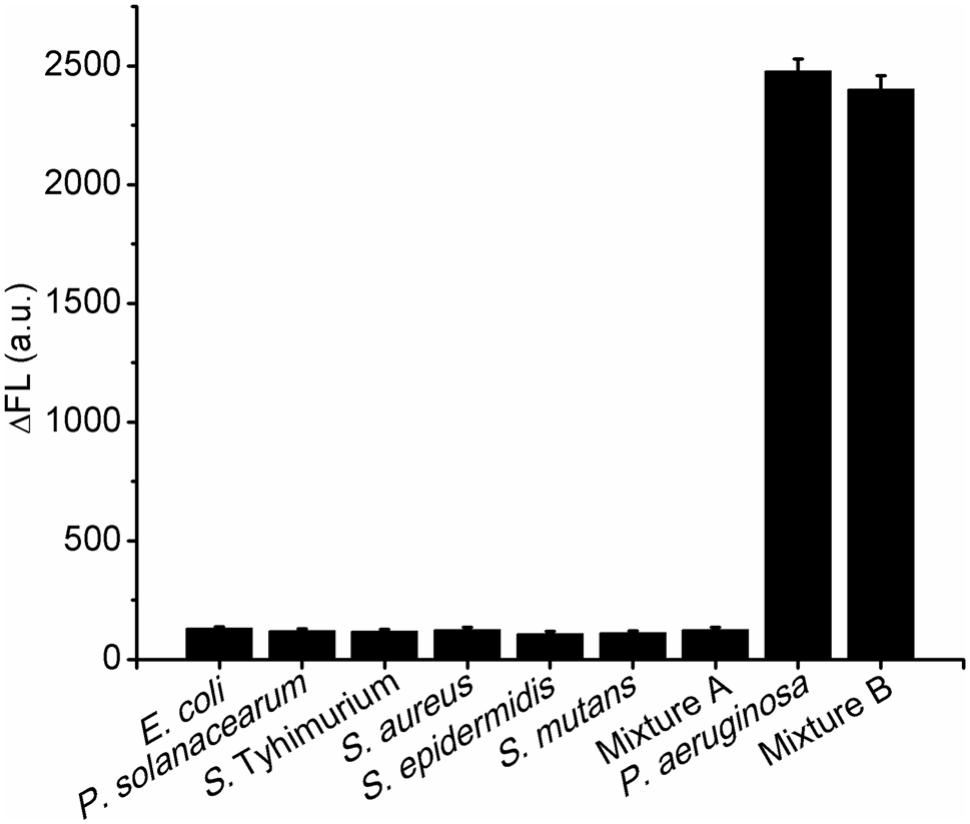
Specificity of RAP for *P. aeruginosa* detection. The concentrations of all the tested bacteria were 1.0 × 10^5^ CFU⋅mL^−1^ (*n* = 4).

As illustrated in Fig. 4, the ID values of the tested interference bacteria were all below 5.26%. For the further investigation of potential interference to *P. aeruginosa* detection, Mixture A was composed of all the six interference bacteria and Mixture B was prepared by mixing *P. aeruginosa* with Mixture A. The ID value of Mixture A was 4.37%. Compared to that of *P. aeruginosa*, the ΔFL intensity of Mixture B showed the minor difference (3.14%).

Twenty five strains of *P. aeruginosa* with different characteristics (Table S1) were tested to investigate the strain specificity of this protocol. As shown in Table S2, the FL signals from the 25 strains of *P. aeruginosa* showed minor differences from -0.78% to 0.66% in comparison with those signals from the host strain. Therefore, this RAP for *P. aeruginosa* detection showed good specificity of species.

### Practical sample detection

To investigate the potential application of this RAP for *P. aeruginosa* detection, 5% glucose injection, rat plasma and human urine were spiked with *P. aeruginosa* suspension at given concentrations. As shown in Table 1, the recovery values ranged from 90.1% to 104.2%, with the RSD all below 5.0%. These results demonstrated the reliability of RAP for detecting *P. aeruginosa* in a complex matrix.

**Table 1:** Recovery tests for *P. aeruginosa* detection spiked in practical samples (*n* = 4).

Sample	Spiked (CFU mL^−1^)	Recovery (%)	RSD (%)
Glucose injection	1.0 × 10^6^	104.2	3.2
	1.0 × 10^5^	95.6	3.1
	1.0 × 10^4^	98.7	2.8
	1.0 × 10^3^	93.4	4.6
Human urine	1.0 × 10^6^	97.6	4.5
	1.0 × 10^5^	98.9	4.7
	1.0 × 10^4^	93.9	2.7
	1.0 × 10^3^	92.2	5.0
Rat plasma	1.0 × 10^6^	101.6	2.1
	1.0 × 10^5^	93.5	3.8
	1.0 × 10^4^	97.2	4.6
	1.0 × 10^3^	90.1	3.9

### AST of P. aeruginosa

AST of *P. aeruginosa* was evaluated by detecting the ΔFL signals of 1.0 × 10^5^ CFU⋅mL^−1^ *P. aeruginosa* cultured with serial concentrations of antibiotics. According to the guidance of CLSI M100S, the four antibiotics of group A including PIP/TAZ, CAZ, TOB and GEN and one antibiotic of group B selected as LVX were utilized to validate the AST of *P. aeruginosa* to demonstrate its reliability. After *P. aeruginosa* was cultured with the absence (blank group, BG) and the presence (test group, TGs) of serial concentrations of antibiotics for 2 h at 37 °C, the ΔFL signals of *P. aeruginosa* were calculated and compared. The same amounts of *P. aeruginosa* suspension stored at 4 °C were detected as the control group (CPs). Since *P. aeruginosa* at 4 °C grew extremely slowly, the concentrations of *P. aeruginosa* was considered as remaining almost unchanged. The results of AST were obtained through comparing the ΔFL signals of TGs with those of CGs and TGs.

**Figure 5:**
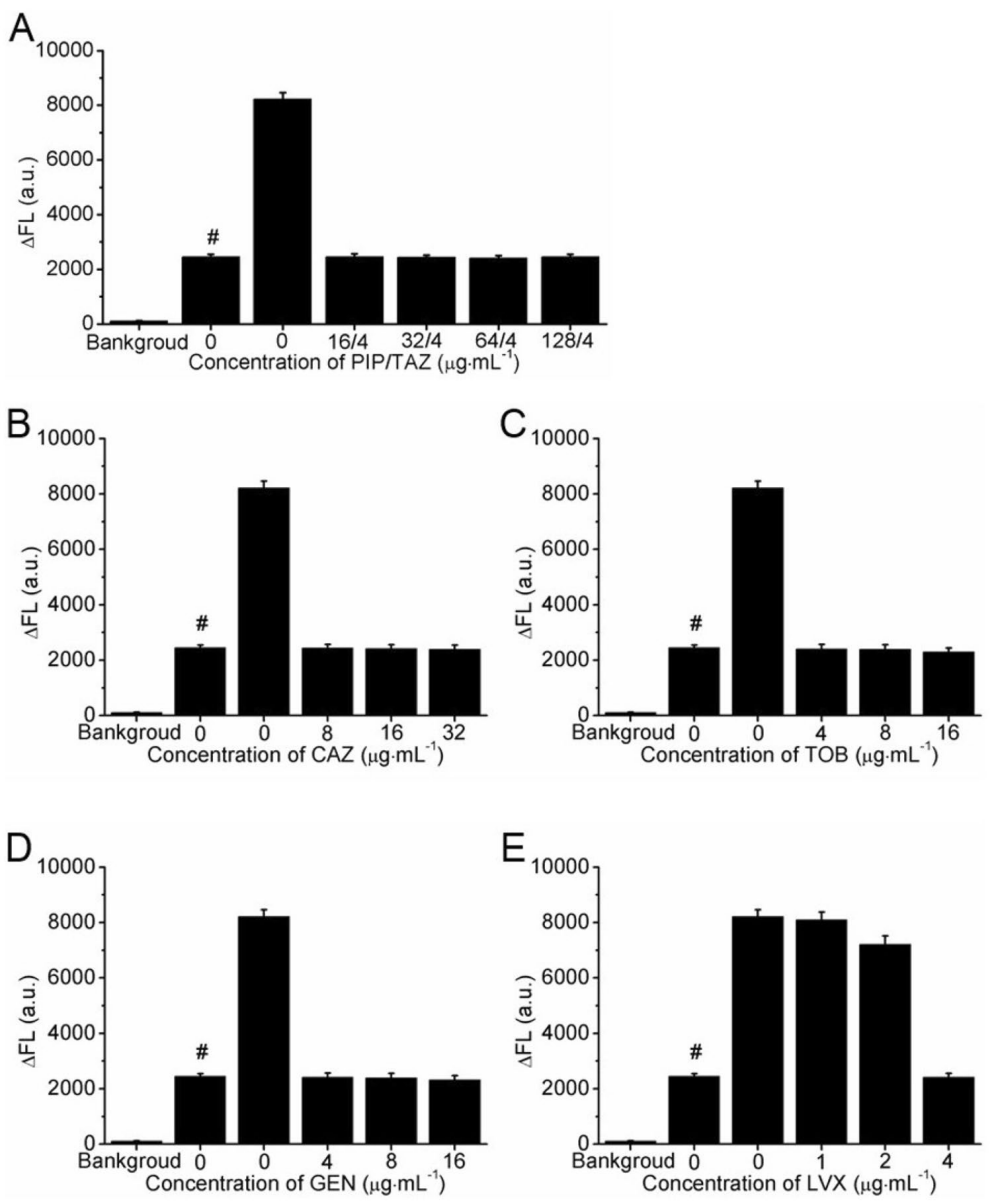
AST of *P. aeruginosa* (ATCC 27,853) treated by (**A**) PIP/TAZ, (**B**) CAZ, (**C**) TOB, (**D**) GEN and (**E**) LVX. Background signals represent the ΔFL from PBS. # signifies the *P. aeruginosa* suspension was kept at 4 °C before the performance of AST (*n* = 4).

For the AST of *P. aeruginosa* to PIP/TAZ, at the concentration ranging from 16/4 to 128/4 μg⋅mL^−1^, the ΔFL signals of TGs were about 99.7% and 29.7% of those of CGs and BGs, respectively (Fig. 5A). As shown in Fig. 5B–D, similar results were also found for CAZ (8–32 μg⋅mL^−1^), TOB (4–16 μg⋅mL^−1^) and GEN (4–16 μg⋅mL^−1^). These results demonstrate that under these antibiotics concentrations the growth of *P. aeruginosa* was significantly inhibited. The minimum inhibitory concentrations (MICs) of PIP/TAZ, CAZ, TOB and GEN were < 16/4, < 8, < 4 and < 4 μg⋅mL^−1^, respectively. According to the guidance of CLSI M100S (Table S3), *P. aeruginosa* was susceptible to these four antibiotics (Table 2). For the AST of *P. aeruginosa* to LVX, when the concentrations were 1 and 2 μg⋅mL^−1^, the ΔFL signals of TGs were about 312% and 93.2% of those of CGs and BGs, respectively (Fig. 5E). These results demonstrated that the growth of *P. aeruginosa* was slightly inhibited by LVX in comparison with the normal growth of *P. aeruginosa* (BGs). However, the concentration of LVX reached 4 μg⋅mL^−1^, the ΔFL signals of TGs reduced to about 99.7% and 29.7% of those of CGs and BGs, respectively. It shown that the growth of *P. aeruginosa* was significantly influenced by LVX at the concentration of 4 μg⋅mL^−1^. Therefore, the MIC of LVX was 4 μg⋅mL^−1^ and *P. aeruginosa* was resistant to LVX (Table 2).

**Table 2:** The AST results of this protocol and CLSI data for *P. aeruginosa* (ATCC 27,853).

Antibiotics		PIP/TAZ	CAZ	TOB	GEN	LVX
Testing results	MIC (μg mL^−1^)	< 16/4	< 8	< 4	< 4	4
	Susceptibility	S	S	S	S	R
CLSI data	Susceptibility	S	S	S	S	I or R

S: susceptible, R: resistant, I: intermediate.

The AST results for all the testing antibiotics were consistent with the provided data of the document of CLSI 100S. These results demonstrated that the RAP protocol showed good reliability for the AST.

### Other bacteriophage-based biosensors for P. aeruginosa detection

As listed in Table 3, several bacteriophage-based biosensors were previously reported for *P. aeruginosa* detection. None of these biosensors were utilized to perform the AST of *P. aeruginosa*. The recognition elements of these biosensors included the living entities of the bacteriophages^28,29^ and the proteins^30^ or peptides^31–34^ displaying on the coat proteins of the bacteriophages. As the recognition elements for *P. aeruginosa* detection, bacteriophage entities offered several advantages such as a relatively cheap and easy production and their stability to pH and temperature variations. However, there still remained a great challenge of immobilization of the bacteriophages on the sensor surface in the right orientation for the optimized capture of *P. aeruginosa*^35^. Another disadvantage of bacteriophage as a whole was the fact that they were biological active and infectious against the host *P. aeruginosa*^35^. TFP as the recognition element, shows ideal innate non-lytic activity for bacteria capture and sample manipulation. Moreover, TFP can also be recombinantly produced for the fixed orientation by expressing biotinylated proteins as the recombinant fusion protein on the N-terminal containing the avi-tag of GLNDIFEAQKIEWHE could be biotinylated specifically at the site of lysine residues (K).

**Table 3:** List of several phage-based biosensors for *P. aeruginosa* detection.

Recognition elements	Transduction platform	Limit of detection	AST	References
Bacteriophage	Electrochemiluminescent	56 CFU mL^−1^	No	Yue et al.^27^
	Real-time PCR	10^2^ CFU mL^−1^	No	Wang et al.^28^
Bacteriophage display peptide	Colorimetric	∼100 cells mL^−1^	No	Peng et al.^29^
	Micro-Raman spectroscopy	10^3^ cells mL^−1^	No	Lentini et al.^30^
	Surface Enhanced Raman Spectroscopy	No mentioned	No	Franco et al.^31^
	Micro-Raman spectroscopy	10 CFU mL^−1^	No	De Plano et al.^32^
	Fluorescence	5 × 10^4^ CFU mL^−1^	No	Pavlyuk et al.^33^
TFP	Fluorescence	33 CFU⋅mL^−1^	Yes	This RAP

## Conclusion

In conclusion, a rapid, sensitive and specific RAP using TFP and magainin II as dual recognition elements was developed to perform the detection and AST of *P. aeruginosa*. Since TFP can specifically recognize the target cells of *P. aeruginosa* from other interference bacteria, the results of AST can actually be obtained within 4 h without the time-consuming process of bacterial isolation and identification, which can facilitate the decreasing frequency of irrational empiric antibiotic therapy. Based on this proof-of-principle work, the detection and AST of other bacteria can be facilely completed by the expression of the TFP of their bacteriophages. In the future work, we will focus on further reduce the detection time of AST based on TFP recognition through other detection technique such as microfluidic system or single-cell imaging.

## Supplementary Information

Supplementary Information.
