# Supplementary material for: Specific and rapid reverse assaying protocol for detection and antimicrobial susceptibility testing of Pseudomonas aeruginosa based on dual molecular recognition
Source: Sci Rep. 2021 May 27;11:11101. doi: 10.1038/s41598-021-90619-3 (PMC8159986; doi:10.1038/s41598-021-90619-3)
Supplement: Supplementary file 1 — Supplementary Information. [file 41598_2021_90619_MOESM1_ESM.doc]

**Electronic supplementary material**

Specific and rapid reverse assaying protocol for detection and antimicrobial susceptibility testing of *Pseudomonas aeruginosa* based on dual molecular recognition

Yong He^1,*^, Hang Zhao^1^, , Yuanwen Liu^1^, He Zhou^2,*^

^1^ Department of Pharmacy, Affiliated Hospital of Zunyi Medical University, Zunyi 563000, China

^2^ Zunyi institute for food and drug control, Zunyi 563000, China

**Table of Contents**

Phage and TFP characteristics and production methodology·····························S3

Figure S1·························································································S7

Figure S2·························································································S8

Figure S3·························································································S9

Figure S4·······················································································S10

Table S1························································································S11

Table S2························································································S12

Table S3························································································S13

**Phage and TFP characteristics**

The bacteriophage of *P. aeruginosa* was isolated from hospital sewage, which has been characterized in the previous work ^[1]^. Briefly, the *P. aeruginosa* bacteriophage with an icosahedral head and a contractile tail is proposed to be classified as a new bacteriophage genus of *Myoviridae*. The bacteriophage has 157 open reading frames (ORFs). Of these, 143 proteins are homologs of known proteins, but only 38 proteins could be functionally identified. Protein 065, 067, 069, 071 and 072 are assumed to be baseplate protein, baseplate related protein, primary tail fiber protein, secondary tail fiber protein and endolysin, respectively. The NCBI reference sequence of the phage and TFP was NC_019913.1 and YP_007236480.1, respectively. TFP’s sequence was NC_019913.1 (42636..44648).

**Preparation of phage template DNA**

Single plaque of phages was added into 200 mL of *P. aeruginosa* PA1 suspension at early logarithmic phase (OD_600_ 0.3~0.4). The suspension was cultured till it turned transparent. Then 11.6 g of NaCl was added into the lysate to completely release phages from cell debris. After the cell debris was removed by centrifugation at 10,000 *g* for 10 min, the obtained supernatant was added with 10% PEG 8,000 to precipitate phages overnight. The precipitated phages were collected by centrifugation at 12,000 *g* for 10 min, followed by purification with equal volume of chloroform. Finally, the phages suspension was stored in PBS at 4 °C.

Forty microliters of 500 mM EDTA, 2.5 μL of 20 µg µL^-1^ proteinase and 50 μL of 10% SDS were added into 900 μL of phages suspension, followed by incubation at 56 °C for 1 h. The obtained suspension was mixed with equal volume of phenol-chloroform-isoamyl alcohol (25:24:1). After 10-min centrifugation at 5,000 *g*, the aqueous layer was collected and extracted with equal volume of chloroform. Subsequently, the obtained aqueous layer was mixed with 600 μL of isopropanol, and then stored at -20 °C overnight. The mixture was centrifuged at 12,000 *g* and 4 °C for 10 min, and the collected precipitated DNA was washed with 70% and 100% ethanol in turn. Finally, the obtained phage DNA was resuspended in 10 mM Tris-HCl buffer (pH 8.0) containing 1.0 mM EDTA and stored at -20 °C till use.

**PCR amplification of TFP gene**

The forward primer and reverse primer for TFP gene were 5’-ATCAT CATAT GAAAA AAAAA GCTGA TTACA GTCAA CTACC TA-3’ and 5’-TGTCT GCGGC CGCAG AAATG CGCTG CCAGA GAGTG-3’, respectively. Amplification of TFP gene was performed in a 50-µL mixture containing 10 µL of 5 × amplification buffer, 5.0 µL of 2.5 mM dNTP, 2.0 µL of forward primer, 2.0 µL of reverse primer, 1.0 µL of *FastPfu* DNA polymerase, 29.5 µL of H_2_O and 0.50 µL of template phage DNA. The PCR reaction conditions were listed as following: initial denaturation at 95 °C for 2 min; 38 cycles of 95 °C for 20 s, 57 °C for 20 s and 72 °C for 20 s; last polymerization at 72 °C for 5 min.

**Construction of recombinant pET21a-TFP gene**

The PCR product of TFP gene and pET21a vector gene were treated with double enzyme digestion using NdeI and NotI. Briefly, 5.0 µL of 10 × Quickcut Green buffer, 5.0 µL of Quickcut NdeI, 5.0 µL of Quickcut NotI, 10 µL of H_2_O and 25 µL of PCR product of TFP gene (or pET21a gene) were mixed and reacted at 37°C for 5 h. After the enzyme-digested products were purified using agarose gel electrophoresis, they were linked together by using T4 DNA ligase in a 10-µL mixture containing 1.0 µL of 10 × buffer, 1.0 µL of PEG 4,000, 0.50 µL of H_2_O, 0.50 µL of T4 DNA ligase, 5.0 µL of pET21a digested fragment and 2.0 µL of TFP digested fragment. After the sequence of pET21a-TFP gene was confirmed, pET21a-TFP gene was transformed into *E. coli* BL21(DE3)pLysS for screening the positive clones.

**TFP expression**

Single colony of the positive clones was cultured in 2,000 mL LB broth containing 50 μg mL^-1^ ampicillin at 37 °C under constant shaking at 180 rpm till OD_600_ reached 0.6. Subsequently, the bacterial culture was added with 20.2 mL of 10 mM IPTG, followed by overnight culture at 16 °C. After centrifugation at 3,000 *g* for 3 min, the collected bacteria were resuspended in 50 mL of pre-cooled NTA buffer, and allowed to stand for 30 min in ice bath. Then the bacteria were disrupted by ultrasonication for 30 min, and the inclusion body of TFP was collected.

**TFP renaturation**

After centrifugation at 16,000 *g* and 16 °C for 5 min, the inclusion body of TFP was resuspended in 45 mL of NTA buffer. The suspension was added with 5.0 mL of 10 mM dithiothreitol, and treated with 30-min ultrasonication. Subsequently, the obtained solution was centrifuged at 16,000 *g* and 4 °C for 10 min. Then the collected precipitation was resuspended in 3.0 mL of 6.0 M guanidine hydrochloride, followed by adding with 3.0 mL of 10 mM dithiothreitol. After the precipitation was dissolved at 37 °C, it was centrifuged at 10,000 *g* and 4 °C for 10 min. The collected supernatant was then mixed with 12.0 mL of 3.0 M guanidine hydrochloride at 4 °C. Afterwards, 200 mL of renaturation buffer was dropped into the mixed solution, followed by continuous stirring for 24 h. The solution was dialyzed against 10 mM PBS and concentrated using PEG 20,000.

**TFP purification**

The obtained renatured TFP solution was filtered through a 0.22-μm filter, and then purified using a nickel-affinity chromatographic column at a flow speed of 1.0 mL/min. The column was eluted using NTA buffers containing 30 mM, 60 mM, 200 mM and 500 mM imidazole in turn. The collected eluted solution was confirmed by sodium dodecyl sulfate polyacrylamide gel electrophoresis. Finally, the TFP solution was dialyzed against 10 mM PBS, and stored at - 20 °C with 20% glycerol.


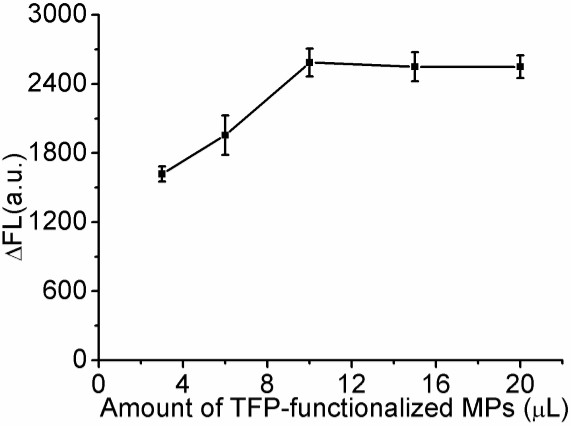


Figure S1. Effects of amount of TFP-functionalized MPs on ΔFL intensity to *P. aeruginosa* at 1.0 🞨10^5^ CFU⋅mL^-1^. All the other experimental conditions were chosen as the optimal conditions (*n* = 4).


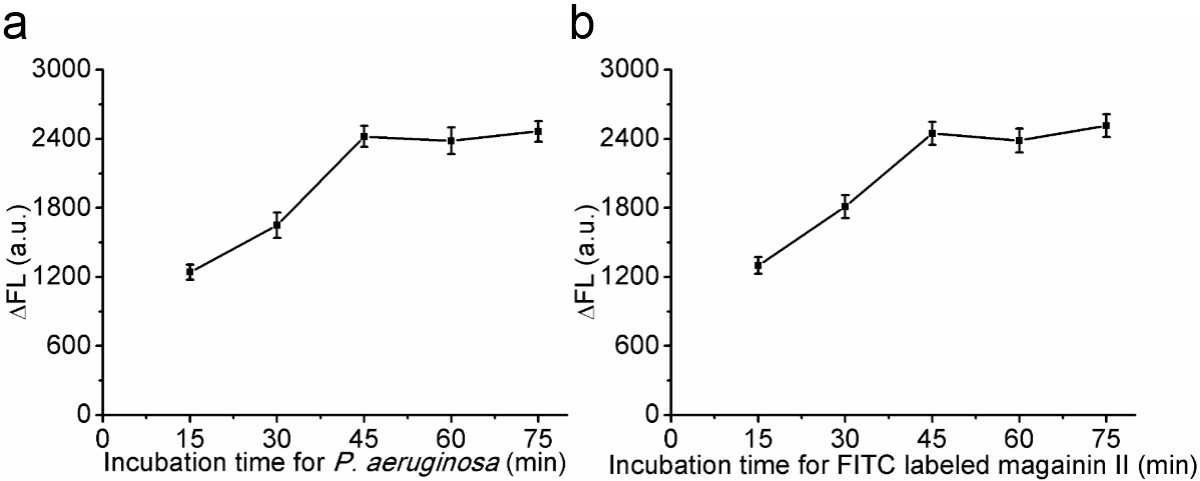


Figure S2. Effects of incubation time (a) *P. aeruginosa* and (b) FITC labeled magainin II on ΔFL intensity to *P. aeruginosa* at 1.0 🞨10^5^ CFU⋅mL^-1^. All the other experimental conditions were chosen as the optimal conditions (*n* = 4).


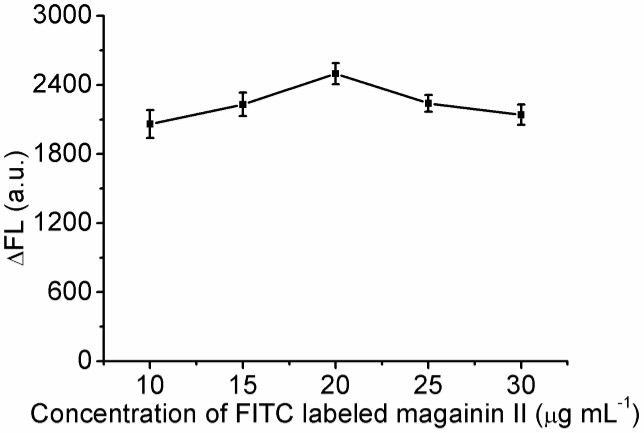


Figure S3. Effects the concentration of FITC labeled magainin II on ΔFL intensity to *P. aeruginosa* at 1.0 🞨10^5^ CFU⋅mL^-1^. All the other experimental conditions were chosen as the optimal conditions (*n* = 4).


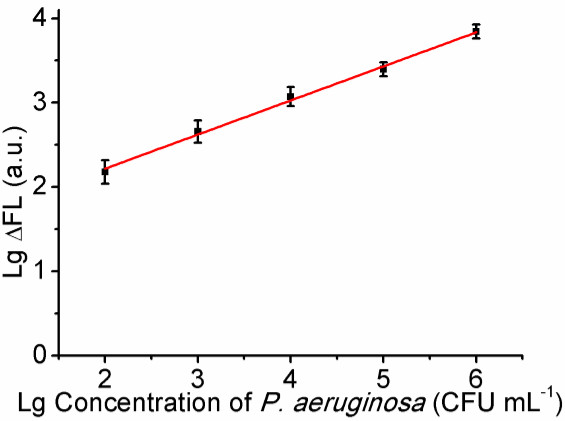


Figure S4. Linear curve of RAP for *aeruginosa* detection. All the other experimental conditions were chosen as the optimal conditions (*n* = 4).

Table S1. List and characteristics of *P. aeruginosa* strains.

| No. | Preservation Number | Source | Size (μm) | G+C content | Other |
| --- | --- | --- | --- | --- | --- |
| 1 | CCTCC KB 20081316 | Xinjiang oilfield | (0.5～0.7)×(1.5～3.0) | 67.2％ | Produce pyocyanine |
| 2 | CCTCC KB 20081317 | Liaoning oilfield | (0.5～0.7)×(1.5～3.0) | 67.2％ | Produce pyocyanine |
| 3 | CCTCC KB 20081318 | Dagang oilfield | (0.5～0.7)×(1.5～3.0) | 67.2％ | Produce pyocyanine |
| 4 | CCTCC KB 20081392 | No mentioned | (0.5～0.7)×(1.5～2.8) | No mentioned | Produce pyocyanine |
| 5 | CCTCC KB 20081393 | No mentioned | (0.5～0.7)×(1.5～2.8) | No mentioned | Produce pyocyanine |
| 6 | CCTCC KB 20081396 | No mentioned | (0.5～0.7)×(1.5～2.8) | No mentioned | Produce pyocyanine |
| 7 | CCTCC KB 20081397 | No mentioned | (0.5～0.7)×(1.5～2.8) | No mentioned | Produce pyocyanine |
| 8 | CCTCC KB 20081398 | No mentioned | (0.5～0.7)×(1.5～2.8) | No mentioned | Produce pyocyanine |
| 9 | CCTCC KB 20081399 | No mentioned | (0.5～0.7)×(1.5～2.8) | No mentioned | Produce pyocyanine |
| 10 | CCTCC KB 20081400 | No mentioned | (0.5～0.7)×(1.5～2.8) | No mentioned | Produce pyocyanine |
| 11 | CCTCC KB 20081401 | No mentioned | (0.5～0.7)×(1.5～2.8) | No mentioned | Produce pyocyanine |
| 12 | CCTCC KB 20082381 | No mentioned | 0.6×2μm | No mentioned | Non-sporing |
| 13 | CCTCC KB 20082384 | No mentioned | 0.6×2μm | No mentioned | Polysaccharide capsule |
| 14 | CCTCC KB 20082416 | No mentioned | 0.6×2μm | No mentioned | Non-sporing |
| 15 | CCTCC KB 20082445 | No mentioned | 0.6×2μm | No mentioned | Polysaccharide capsule |
| 16 | CCTCC KB 20082544 | No mentioned | (0.5～0.8)×(1.5～3.0) | No mentioned | Alive at 42°C |
| 17 | CCTCC KB 20082584 | No mentioned | Different length | No mentioned | Raw ginger smelling |
| 18 | CCTCC DB 20082415 | SDMCC | Different length | No mentioned | Produce pyocyanine |
| 19 | CCTCC DB 20082436 | ATCC | Different length | No mentioned | Non-encapsulated |
| 20 | CCTCC AB 2013185 | Wuhan university | (0.5～2.0)×(1.5～5.0) | No mentioned | Non-sporing |
| 21 | CCTCC AB 2010174 | Kavanagh Merck | (0.5～0.8)×(1.5～3.0) | No mentioned | Raw ginger smelling |
| 22 | CCTCC AB 93078 | Shanxi | Different length | No mentioned | Non-sporing |
| 23 | CCTCC HB 20082566 | No mentioned | (0.5～0.8)×(1.5～4.0) | No mentioned | Non-sporing |
| 24 | CCTCC AB 2013184 | Wuhan university | (0.5～1.0)×(1.5～5.0) | No mentioned | Non-encapsulated |
| 25 | CCTCC AB 2010470 | India | Different length | No mentioned | Produce fluorescence |

Table S2. Strain specificity of RAP for *P. aeruginosa* detection. The concentrations of all the *P. aeruginosa* strains were 1.0 × 10^5^ CFU⋅mL^−1^ (*n* = 4).

| No. | Preservation Number | *ΔFL* (a. u.) | RSD | Difference to the host strain |
| --- | --- | --- | --- | --- |
| 1 | CCTCC KB 20081316 | 2471.3 | 2.66% | 0.07% |
| 2 | CCTCC KB 20081317 | 2460.2 | 3.08% | -0.38% |
| 3 | CCTCC KB 20081318 | 2485.4 | 3.43% | 0.64% |
| 4 | CCTCC KB 20081392 | 2455.9 | 3.05% | -0.55% |
| 5 | CCTCC KB 20081393 | 2478.2 | 4.04% | 0.35% |
| 6 | CCTCC KB 20081396 | 2450.2 | 3.66% | -0.78% |
| 7 | CCTCC KB 20081397 | 2467.8 | 2.59% | -0.07% |
| 8 | CCTCC KB 20081398 | 2455.6 | 3.05% | -0.56% |
| 9 | CCTCC KB 20081399 | 2475.3 | 4.49% | 0.23% |
| 10 | CCTCC KB 20081400 | 2469.8 | 3.04% | 0.01% |
| 11 | CCTCC KB 20081401 | 2471.6 | 3.60% | 0.09% |
| 12 | CCTCC KB 20082381 | 2481.7 | 3.69% | 0.49% |
| 13 | CCTCC KB 20082384 | 2477.7 | 3.77% | 0.33% |
| 14 | CCTCC KB 20082416 | 2468.2 | 3.62% | -0.05% |
| 15 | CCTCC KB 20082445 | 2478.4 | 3.05% | 0.36% |
| 16 | CCTCC KB 20082544 | 2477.5 | 2.81% | 0.32% |
| 17 | CCTCC KB 20082584 | 2458.6 | 4.88% | -0.44% |
| 18 | CCTCC DB 20082415 | 2468.7 | 6.45% | -0.03% |
| 19 | CCTCC DB 20082436 | 2455.5 | 4.72% | -0.57% |
| 20 | CCTCC AB 2013185 | 2480.1 | 4.04% | 0.43% |
| 21 | CCTCC AB 2010174 | 2475.9 | 4.68% | 0.26% |
| 22 | CCTCC AB 93078 | 2474.9 | 5.24% | 0.22% |
| 23 | CCTCC HB 20082566 | 2470.3 | 3.18% | 0.03% |
| 24 | CCTCC AB 2013184 | 2459.9 | 3.48% | -0.39% |
| 25 | CCTCC AB 2010470 | 2485.7 | 3.93% | 0.66% |
| 26 | The host strains | 2469.5 | 4.51% | 0 |

Table S3. MIC interpretive standards for *P. aeruginosa*.

| Report Group | Antibiotic | MIC Interpretive Criteria(μg mL^-1^) | | |
| --- | --- | --- | --- | --- |
|  |  | S | I | R |
| A | PIP/TAZ | ≤16/4 | 32/4-64/4 | ≥128/4 |
| A | CAZ | ≤8 | 16 | ≥32 |
| A | GEN | ≤4 | 8 | ≥16 |
| A | TOB | ≤4 | 8 | ≥16 |
| B | LVX | ≤2 | 4 | ≥8 |

Reference

1. Lu, S. G. et al. Genomic and proteomic analyses of the terminally redundant genome of the *Pseudomonas aeruginosa* phage PaP1: establishment of genus PaP1-like phages. *Plos one* **8**, e62933 (2013).
